# Global Prevalence and Drivers of Dental Students’ COVID-19 Vaccine Hesitancy

**DOI:** 10.3390/vaccines9060566

**Published:** 2021-05-29

**Authors:** Abanoub Riad, Huthaifa Abdulqader, Mariana Morgado, Silvi Domnori, Michal Koščík, José João Mendes, Miloslav Klugar, Elham Kateeb, on behalf of IADS-SCORE

**Affiliations:** 1 Department of Public Health, Faculty of Medicine, Masaryk University, 62500 Brno, Czech Republic; koscik@med.muni.cz (M.K.); klugar@med.muni.cz (M.K.); 2 International Association of Dental Students (IADS), 1216 Geneva, Switzerland; vpsr@iads-web.org (H.A.); mmorgado@egasmoniz.edu.pt (M.M.); vppr@iads-web.org (S.D.); 3 Czech National Centre for Evidence-Based Healthcare and Knowledge Translation (Cochrane Czech Republic, Czech EBHC: JBI Centre of Excellence, Masaryk University GRADE Centre), Institute of Biostatistics and Analyses, Faculty of Medicine, Masaryk University, 62500 Brno, Czech Republic; 4 Clinical Research Unit (CRU), Egas Moniz Cooperativa de Ensino Superior, 2829-511 Almada, Portugal; jmendes@egasmoniz.edu.pt; 5 Oral Health Research and Promotion Unit, Faculty of Dentistry, Al-Quds University, Jerusalem 51000, Palestine; ekateeb@staff.alquds.edu; 6 Public Health Committee, World Dental Federation (FDI), 1216 Geneva, Switzerland

**Keywords:** COVID-19 vaccines, cross-sectional studies, decision making, dental education, dental students, international association of dental students, mass vaccination, multicentre study, social determinants of health

## Abstract

Background: Acceleration of mass vaccination strategies is the only pathway to overcome the COVID-19 pandemic. Healthcare professionals and students have a key role in shaping public opinion about vaccines. This study aimed to evaluate the attitudes of dental students globally towards COVID-19 vaccines and explore the potential drivers for students’ acceptance levels. Methods: A global cross-sectional study was carried out in February 2021 using an online questionnaire. The study was liaised by the scientific committee of the International Association of Dental Students (IADS), and data were collected through the national and local coordinators of IADS member organizations. The dependent variable was the willingness to take the COVID-19 vaccine, and the independent variables included demographic characteristics, COVID-19-related experience, and the drivers of COVID-19 vaccine-related attitude suggested by the WHO SAGE. Results: A total of 6639 students from 22 countries, representing all world regions, responded to the questionnaire properly. Their mean age was 22.1 ± 2.8 (17–40) years, and the majority were females (70.5%), in clinical years (66.8%), and from upper-middle-income economies (45.7%). In general, 22.5% of dental students worldwide were hesitant, and 13.9% rejected COVID-19 vaccines. The students in low- and lower-middle-income (LLMI) economies had significantly higher levels of vaccine hesitancy compared to their peers in upper-middle- and high-income (UMHI) economies (30.4% vs. 19.8%; *p* < 0.01). Conclusions: The global acceptance level of dental students for COVID-19 vaccines was suboptimal, and their worrisome level of vaccine hesitancy was influenced by the socioeconomic context where the dental students live and study. The media and social media, public figures, insufficient knowledge about vaccines, and mistrust of governments and the pharmaceutical industry were barriers to vaccination. The findings of this study call for further implementation of epidemiology (infectious diseases) education within undergraduate dental curricula.

## 1. Introduction

Vaccine hesitancy (VH) is defined by the World Health Organization (WHO) Strategic Advisory Group of Experts on Immunization (SAGE) as a “delay in acceptance or refusal of vaccination despite the availability of vaccination services” [1]. Given the urgency of mass vaccination against severe acute respiratory syndrome coronavirus 2 (SARS-CoV-2) strategies, VH is increasingly recognized as a serious public health threat that requires thorough investigation among various population groups to fully understand its drivers as well as its prevalence [2,3].

However, healthcare workers (HCWs) are usually perceived by their patients as the most reliable information source about vaccines; this group sometimes shows different levels of VH [4,5]. Karafillakis et al. (2016) found that the fear of vaccine side effects, apprehension of new vaccines due to lack of safety data, and mistrust of pharmaceutical companies due to financial interests were the most prominent drivers of VH among HCWs in Europe [6]. Unfortunately, all these issues are relevant to the context of coronavirus disease 2019 (COVID-19) vaccines recently produced through intensified manufacturing processes and used through emergency use authorization (EUA) [7].

Dental students represent a particular subset of the healthcare students’ population who will play a key role in shaping their patients’ health-related attitudes and behaviours in the near future, as they are perceived as role models of a healthy lifestyle [8]. Adopting positive health-related beliefs and attitudes by HCWs increases their preparedness and capacity to counsel patients on behavioural changes [9]. Therefore, self-care is a core competency of medical education and a cost-effective public health policy for sustainable health promotion [10,11]. Moreover, medical students retain the highest levels of health-related knowledge and attitudes, making them the opinion leaders of public health issues among the university students’ community [12].

Additionally, dental students differ from the general university student population because they are prone to an increased risk of contracting infectious diseases as part of their clinical training requirement [13]. Therefore, they are obliged to receive certain vaccines prior to joining the clinical years of their undergraduate courses, including hepatitis B, tetanus, and influenza vaccines [14]. These aggressive policies had led to a significant increase in immunized dentists against a wide array of occupational infections during recent years in some low- and low-middle-income economies [15,16]. Nevertheless, dental students’ knowledge and attitudes towards the relatively novel and nonmandatory vaccines, such as the human papillomavirus (HPV) vaccine, remained suboptimal in countries of various economic capacities, e.g., India, Saudi Arabia, and the United States of America (USA) [17,18,19].

Clinical-year dental students were found to have substantially higher levels of positive attitudes and knowledge towards HPV and hepatitis B vaccines, respectively, compared to their pre-clinical peers [19,20]. Farsi et al. (2020) had found that female dental students showed a significantly higher level of HPV vaccine acceptance than their male peers [19]. Similarly, Rutkoski et al. (2020) found that female dental students were more knowledgeable about the HPV vaccine [18]. The conspiracy beliefs and misconception about the immunity system played a decisive role in decreasing the university students’ acceptance level of the COVID-19 vaccine [21]. However, there is a lack of worldwide evidence on COVID-19 VH levels and their association with the economy; the indirect evidence suggests that economic hardship is a clear determinant of VH among individuals in the same country [22]. In a recent Italian cross-sectional study, the recovered COVID-19 patients showed substantial levels of VH towards the COVID-19 vaccine (59.2%) and influenza vaccine (54.6%) [23]. Older age and previous 2019 influenza shots were the main predictors of positive attitudes towards vaccination [23].

Social media platforms play a key role in communicating health-related information, including pro- and anti-vaccination messages, which can affect public opinion about vaccines, their effectiveness, and safety [24]. A recent cross-sectional study revealed that mistrust of government was strongly correlated with vaccine hesitancy among the Austrian population; thus, suggesting that disengagement from public discourse endangers vaccination strategies [25]. Religious and cultural values can also undermine the willingness to receive the vaccines; therefore, there are emerging calls for urgent interventions to confront misconceptions about vaccines through religious institutions [26]. Therefore, WHO SAGE acknowledged social media, mistrust of government and pharmaceutical industry, and personal beliefs as contextual drivers of vaccine hesitancy [2]. In addition, general attitudes towards newly developed vaccines, knowledge about vaccine safety and effectiveness, and availability of vaccines are depicted by the WHO SAGE as personal/social and vaccine-specific drivers of VH [2].

The primary objective of this study was to estimate the prevalence of COVID-19 VH among a global sample of dental students. The secondary objectives were to explore the potential drivers for VH of dental students and to evaluate their impact on students’ acceptance of the COVID-19 vaccine.

## 2. Materials and Methods

### 2.1. Study Design

A cross-sectional survey-based study was carried out between the 6th and 28th February 2021 by the national and local member organizations of the International Association of Dental Students (IADS) [27]. The study utilized an online self-administered questionnaire (SAQ) of multiple-choice items developed through KoBoToolbox (Harvard Humanitarian Initiative, Cambridge, MA, USA, 2021) [28].

After ethical clearance, invitation emails were sent to all the national and local delegates of IADS to participate in this cross-sectional study aiming to evaluate the attitudes of dental students worldwide towards the COVID-19 vaccine. Online orientation sessions were held to demonstrate the study objectives and the role of national and local coordinators in this study who were primarily concerned with survey dissemination and data collection. The Standing Committee on Research and Education (SCORE) of IADS was in charge of supervising the whole project and facilitating communication among the national and local coordinators [29]. The study was conducted and reported according to the Strengthening the Reporting of Observational Studies in Epidemiology (STROBE) statement for cross-sectional studies [30].

### 2.2. Participants

The target population of this study was the undergraduate dental students in the participating countries. Given the global variability of dental education systems, students of the compulsory training year (dental interns) and the students who graduated within the last 12 months were included. The participation in this study was voluntary, and the participants were not financially compensated and received no other means of incentives to limit selection bias. All participants provided their digital informed consent prior to filling in the questionnaire, and they were offered to withdraw from the study at any moment before submitting their answers without justification.

The national and local coordinators used various methods to circulate the uniform resource locator (URL) of the questionnaire, including social media platforms (Facebook, Twitter, and Instagram), instant messaging groups (WhatsApp and WeChat), and distribution lists of their organizations and universities.

The pragmatic sample size of participating countries was calculated using Epi-Info^TM^ version 7.2.4 (CDC, Atlanta, GA, USA, 2020) according to the total number of dental students in each participating country and assuming 50% of outcome probability with a confidence level of 95% and an error margin of 5% [31] (Supplementary File, Table S1).

A total of 6680 students participated in this study, with 41 students having filled in the questionnaire improperly; therefore, they were excluded from the final analyses.

### 2.3. Instrument

The SAQ consisted of 20 multiple-choice items which required on average 7–9 min to be completed, and it was divided into four categories: (a) demographic data, including gender, age, academic level, and country; (b) COVID-19-related experience, including previous infection, providing care to a COVID-19 patient, having a COVID-19 patient within the social circle, and having a deceased COVID-19 patient within the social circle; (c) willingness to take the COVID-19 vaccine determined by a 5-point Likert scale; and (d) the drivers of COVID-19 vaccine-related attitude (Supplementary File; Table S2).

The items of the fourth category were adopted from the compendium validated by the WHO SAGE [2]. The impact of media/social media, influential leaders and gatekeepers, trust in government, trust in pharmaceutical companies, and personal, religious, and cultural values were selected as the contextual drivers, while the beliefs of health and natural immunity, and perceived knowledge sufficiency were depicted as the individual drivers. Out of the vaccine-specific drivers’ group, the risk to benefit ratio of administering the COVID-19 vaccine, introducing a new vaccine, and the vaccine availability were selected. The items were selected by a panel of experts in medical education and public health, the selection criteria were the relevance of the items to the COVID-19 vaccines context [2].

The experts’ panel reviewed the appropriateness and clarity of the suggested questionnaire to determine its content validity. Consequently, 18 dental students were recruited to fill in the questionnaire twice, with a minimum interval of 48 h to evaluate its reliability. The mean Cohen’s kappa coefficient of the test re-test was 81.83 ± 0.16 (0.55–1), indicating that the questionnaire retained a perfect level of reliability (Table 1).

### 2.4. Ethical Considerations

The study protocol was reviewed and approved by the Ethics Committee of the Faculty of Medicine, Masaryk University (MUNI) on 20th January 2021 with reference No. 4/2021. The questionnaire collected no identifying personal data from the participants. The study data were collected and managed by MUNI in full compliance with the European General Data Protection Regulation 2016/679 (GDPR) [33].

### 2.5. Statistical Analysis

The Statistical Package for the Social Sciences (SPSS) version 27 (SPSS Inc. Chicago, IL, USA, 2020) was used to perform all the statistical tests [34]. Primarily, descriptive analysis was performed for the demographic variables, COVID-19-related experience, willingness to take the COVID-19 vaccine, and the drivers of COVID-19 vaccine-related attitude represented by frequencies, percentages, cumulative percentages, means, and standard deviations. Consequently, the students were classified according to their academic level as pre-clinical (1st year and 2nd year) and clinical (3rd year–fresh graduate). The participating countries were also stratified into four groups according to the latest ranking of the World Bank (the fiscal year 2021) as low-income economies, lower-middle-income economies, upper-middle-income economies, and high-income economies [35]. Inferential statistics were carried out to evaluate the association of the COVID-19 vaccine acceptance level and demographic variables using the Mann–Whitney U-test and the Kruskal–Wallis test. Similarly, the vaccine acceptance level and its associated drivers were evaluated using the Mann–Whitney U-test with a confidence level of 95% and significance value (*p*) ≤ 0.05.

## 3. Results

### 3.1. Demographic Characteristics

Out of the 6639 included students, 4682 (70.5%) were females, 1836 (27.7%) were males, 53 (0.8%) were non-binary, and 68 (1%) preferred not to disclose their gender. The mean age of participants was 22.06 ± 2.79 (17–40) years. While 2206 (33.2%) participants were pre-clinical students, 4433 (66.8%) were in clinical years.

The participating students were from 22 countries: 57 (0.9%) from Albania, 183 (2.8%) from Canada, 169 (2.5%) from Croatia, 381 (5.7%) from Ecuador, 78 (1.2%) from Estonia, 416 (6.3%) from Indonesia, 388 (5.8%) from Iran, 354 (5.3%) from Iraq, 492 (7.4%) from Italy, 129 (1.9%) from Latvia, 107 (1.6%) from Lebanon, 291 (4.4%) from Lithuania, 350 (5.3%) from Malaysia, 153 (2.3%) from Nepal, 379 (5.7%) from Pakistan, 417 (6.3%) from Palestine, 389 (5.9%) from Portugal, 596 (9%) from Russia, 467 (7%) from Sudan, 283 (4.3%) from Tunisia, 386 (5.8%) from Turkey, and 174 (2.6%) from the USA.

According to the latest ranking of the World Bank (the fiscal year 2021), 467 (7%) of the participants were from low-income economies, 1232 (18.6%) from lower-middle-income economies, 3035 (45.7%) from upper-middle-income, and 1905 (28.7%) from high-income economies [35]. According to the World Dental Federation (FDI) geographical categorization, 750 (11.3%) were from Africa, 738 (11.1%) from the Americas, 1298 (19.6%) from Asia-Pacific, 1266 (19.1%) from Eastern Mediterranean, and 2587 (39%) from Europe [36] (Table 2).

### 3.2. COVID-19-Related Experience

A total of 1105 (16.6%) students reported that they had been previously infected by severe acute respiratory syndrome coronavirus 2 (SARS-CoV-2). The students from low- and lower-middle-income economies (LLMI) were significantly (*χ^2^* = 13.81; *p* < 0.01) more infected by COVID-19 compared to the students from upper-middle- and high-income economies (UMHI), 19.5% vs. 15.6% respectively. While 538 (31.7%) students from LLMI provided care to COVID-19 patients, 1270 (25.7%) students from UMHI provided such care with a statistically significant difference (*χ^2^* = 22.64; *p* < 0.01).

The same pattern was found with having COVID-19 patients within students’ social circles, as 1515 (89.2%) students from LLMI versus 4286 (86.8%) students from UMHI knew personally COVID-19 patients (*χ^2^* = 6.65; *p* = 0.01); with having deceased COVID-19 patients within students’ social circles, as 945 (55.6%) students from LLMI versus 2086 (42.2%) students from UMHI reported to having known someone who died from COVID-19 infection (*χ^2^* = 91.41; *p* < 0.01) (Table 3).

The clinical students were significantly more affected by the COVID-19 pandemic than their preclinical peers, as 17.6% vs. 14.7% were previously infected (*χ^2^* = 9.12; *p* = 0.003), 28.1% vs. 25.5% provided care to COVID-19 patients (*χ^2^* = 5.15; *p* = 0.023), 88.9% vs. 84.2% knew personally COVID-19 patients (*χ^2^* = 29.78; *p* < 0.01), and 47.4% vs. 42.2% knew someone who died from COVID-19 infection (*χ^2^* = 15.86; *p* < 0.01).

Male students were significantly (*χ^2^* = 22.67; *p* < 0.01) more infected by COVID-19 compared to their female peers, 19.8% vs. 14.9% respectively. Moreover, 584 (31.8%) males versus 1170 (25%) females provided care to COVID-19 patients (*χ^2^* = 31.18; *p* < 0.01). Contrarily, female students knew personally COVID-19 patients more than male students, 87.8% vs. 86.8% respectively (*χ^2^* = 1.26; *p* = 0.268). Similarly, more females knew deceased COVID-19 patients, 45.9% vs. 45.1% (*χ^2^* = 0.32; *p* = 0.57).

The highest countries with previously infected students were Albania (43.9%), Iran (34.3%), Tunisia (31.1%), Lebanon (29%), Ecuador (24.9%), Russia (23.7%), and the USA (20.1%). The highest countries with students providing care to COVID-19 patients were Iran (49.7%), Albania (49.1%), Tunisia (42%), Iraq (38.4%), Lebanon (36.4%), Palestine (35.3%), and Russia (33.4%).

### 3.3. COVID-19 Vaccine-Related Attitudes

Regarding their attitudes towards the COVID-19 vaccine, 491 (7.4%) students totally disagreed with taking the vaccine, 434 (6.5%) disagreed, 1494 (22.5%) were hesitant about taking the vaccine, 1495 (22.5%) agreed, and 2725 (41%) totally agreed (Table 4).

The LLMI students were significantly (*χ^2^* = 82.26; *p* < 0.01) more hesitant to take the vaccine than their UMHI peers, 30.4% vs. 19.8%, respectively. The highest percentage of hesitant students were from low-income economies (37.5%), followed by lower-middle-income economies (27.8%), upper-middle-income economies (25.2%), and high-income economies (11.1%). Similarly, the highest percentage of resistant students were from low-income economies (18.6%), and the lowest percentage was from high-income economies (7.3%). Contrarily, the lowest agreement level was in low-income economies (43.9%), followed by lower-middle-income economies (56.9%), upper-middle-income economies (58%), and high-income economies (81.6%).

### 3.4. Drivers of COVID-19 Vaccine-Related Attitude

The LLMI students were more significantly (*U* = 3,436,031; *p* < 0.01) influenced by the reports they heard and read in media and social media compared to their UMHI peers, 42% vs. 30.4%, respectively. They were also more significantly (*U* = 3,832,308; *p* < 0.01) influenced by celebrities, religious and political leaders, 21.3% vs. 14.5%.

In terms of confidence in government and pharmaceutical companies, the UMHI students (37.9% and 51% respectively) were more significantly (*U* = 3,608,480, 3,526,701; *p* < 0.01, <0.01 respectively) confident than their LLMI peers (27.1% and 37% respectively). While the UMHI students were more aware of people with religious and cultural values who retained them from taking vaccines, 25% vs. 17% respectively, the LLMI students were more agreeable with this antivaccination stand, 18% vs. 10.9% respectively.

Regarding the individual drivers, 38.1% of LLMI students thought that there was better ways to prevent COVID-19 than using vaccines versus 22.4% of UMHI students (*U* = 3,514,137; *p* < 0.01). Additionally, the UMHI students were significantly (*U* = 3,707,403; *p* < 0.01) more confident that they had enough information about COVID-19 vaccines and their safety compared to their LLMI peers, 33.1% vs. 27%, respectively.

All the vaccine-specific drivers were significantly in favour of UMHI, as 54.4% of UMHI students had higher levels of beliefs that the benefits of COVID-19 vaccines outweigh their reported side effects compared to only 40.2% of LLMI students. Moreover, 45.2% of UMHI students were more inclined to take a newly introduced vaccine versus 37.6% of LLMI students. Similarly, 43.2% of UMHI students felt confident that their health centres would have the COVID-19 vaccine whenever needed compared to only 33.6% of LLMI students (Table 5).

Males had significantly higher levels of belief of natural immunity superiority over vaccines and perceived knowledge sufficiency (*U* = 4,139,866, 3,758,515; *p* = 0.01, <0.01 respectively). Females had lower levels of confidence in government (34.6% vs. 37.4%), confidence in pharmaceutical companies (47.5% vs. 47.9%), belief that COVID-19 benefits outweigh its reported side effects (50.1% vs. 53.5%), and inclination to take newly introduced vaccines (42.1% vs. 46.8%). Across academic levels, the fresh graduates were the least informed about COVID-19 vaccine safety, and they were the most supportive of the belief of natural immunity superiority over vaccines.

### 3.5. Demographic Determinants of COVID-19 Vaccine-Related Attitude

The acceptance level of the COVID-19 vaccine ranged between one denoting “totally disagree”, and five denoting “totally agree”. The mean acceptance level of males was significantly (*U* = 4,165,947; *p* = 0.04) higher than females; it was also higher than non-binary students (3.47 ± 1.42) and the students who did not disclose their gender (3.44 ± 1.43).

The fifth-year students were the most accepting (3.93 ± 1.25); however, the interns were the least accepting (3.59 ± 1.26). Overall, the clinical students were significantly (*U* = 4,655,705; *p* = 0.01) more accepting compared to the pre-clinical students (Table 6).

**Figure 1:**
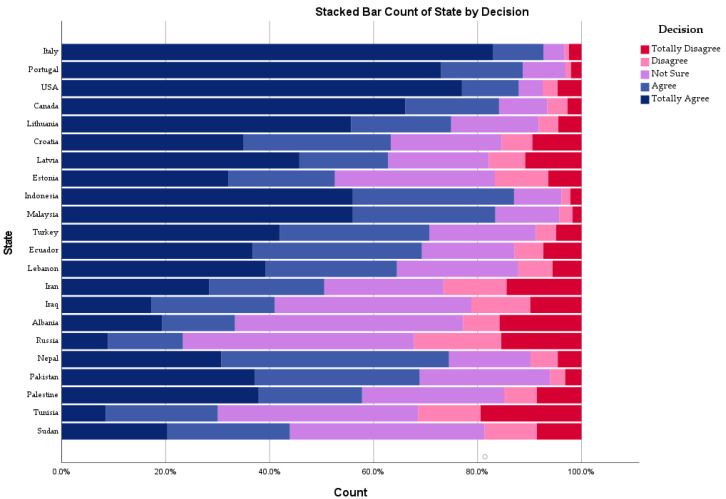
Dental students’ COVID-19 vaccine acceptance level by state, February 2021.

In the high-income group, Italy was the most accepting country (4.7 ± 0.8), followed by Portugal (4.57 ± 0.85) and the USA (4.53 ± 1.04), while Estonia was the least accepting one (3.62 ± 1.22). In the upper-middle-income group, Indonesia was the most accepting country (4.37 ± 0.89), followed by Malaysia (4.33 ± 0.91) and Turkey (3.99 ± 1.11), while Russia was the least accepting one (2.85 ± 1.12). Pakistan was the most accepting lower-middle-income country (3.97 ± 1.01), and Tunisia was the least accepting lower-middle-income country (2.88 ± 1.2) (Figure 1).

**Figure 2:**
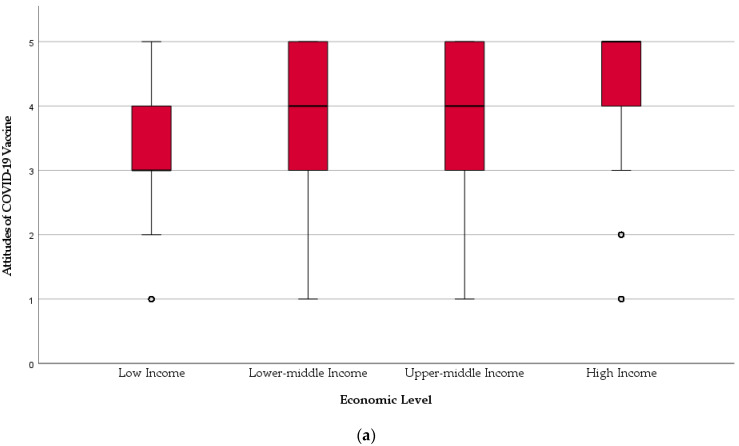

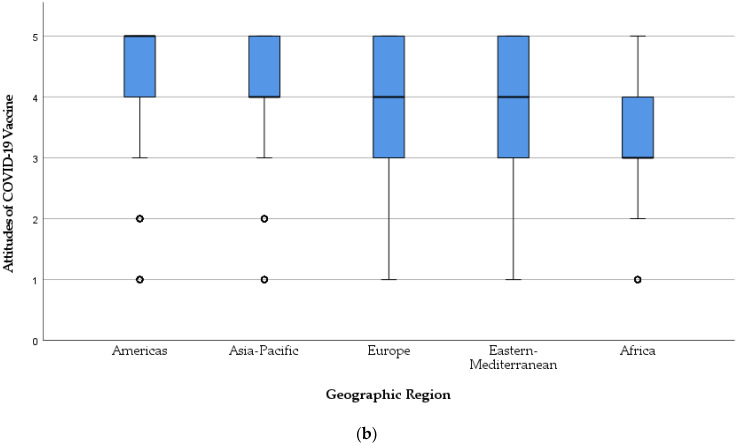
Dental students’ COVID-19 vaccine acceptance by (**a**) economic level and (**b**) geographic region, February 2021.

Africa was the least accepting region (3.18 ± 1.2), followed by Eastern Mediterranean (3.5 ± 1.29), and the Americas was the most accepting region (4.15 ± 1.15). Across the economic ranking, the vaccine acceptance level was gradually distributed with the least accepting being the low-income group (3.37 ± 1.17), and the most accepting being the high-income group (4.36 ± 1.07). The difference between the lower-middle-income group (3.63 ± 1.23) and upper-middle-income group (3.66 ± 1.25) was slight and statistically insignificant (*U* = 1,833,237; *p* = 0.302) (Figure 2).

Regarding their COVID-19-related experience, the students who had been previously infected were significantly (*U* = 2,677,855; *p* < 0.01) less accepting (3.57 ± 1.36), also the students who provided care to COVID-19 patients were significantly (*U* = 4,004,539; *p* < 0.01) less accepting (3.68 ± 1.31). However, having a COVID-19 patient within students’ social circles was significantly (*U* = 2,219,499; *p* < 0.01) associated with a higher acceptance level of COVID-19 vaccine (3.86 ± 1.24), and having a deceased COVID-19 patient within students’ social circles was significantly (*U* = 5,175,777; *p* < 0.01) lowering the COVID-19 vaccine acceptance (3.77 ± 1.26) (Table 7).

### 3.6. Drivers of Dental Students’ COVID-19 Vaccine Acceptance

#### 3.6.1. Contextual Drivers

Globally, the dependence on media and social media to inform vaccine-related decision was significantly (*U* = 2,678,371; *p* < 0.01) associated with a decreased level of vaccine acceptance (3.71 ± 1.21). Similarly, the reliance on public figures and opinion leaders was insignificantly (*U* = 2,495,719; *p* = 0.24) associated with a lower level of vaccine acceptance (3.82 ± 1.27).

The confidence in government was significantly (*U* = 1,467,912; *p* < 0.01) associated with a higher level of vaccine acceptance (4.29 ± 1.10), also the confidence in pharmaceutical companies improved vaccine acceptance (4.31 ± 1.06) significantly (*U* = 1,041,773; *p* < 0.01). The students who agreed with the antivaccination stand based on cultural and religious values had significantly (*U* = 43,532; *p* < 0.01) much lower levels of vaccine acceptance (2.75 ± 1.44).

#### 3.6.2. Individual Drivers

The students who thought COVID-19 should be better prevented by natural immunity than by vaccines had a significantly (*U* = 1,698,349; *p* < 0.01) lower level of vaccine acceptance (3.35 ± 1.38). Additionally, the students with insufficient knowledge about COVID-19 vaccine safety had a significantly (*U* = 1,766,800; *p* < 0.01) lower level of vaccine acceptance (3.48 ± 1.19).

#### 3.6.3. Vaccine-Specific Drivers

The COVID-19 vaccine acceptance level was significantly decreased in the students who did not think that the vaccine’s benefits outweigh its reported side effects, the students who were not inclined to take newly introduced vaccines, and the students who were not confident in finding the vaccine in their local health centre when needed (*U* = 1,010,578, 974,557, 1,608,552; *p* < 0.01, <0.01, <0.01) respectively (Table 8).

## 4. Discussion

Globally, 22.5% of dental students within our sample were hesitant about taking COVID-19 vaccines. Verger et al. (2021) found that 28.39% of healthcare workers in France and French-speaking parts of Belgium and Canada were hesitant about COVID-19 vaccines [37]. The findings of Verger et al. (2021) were a bit close to what had been found in Malta (22%), Portugal (21%), Germany (20%), the Netherlands (19%), and Italy (19%) [38,39]. On the other hand, the studies that sampled general university students showed a wide range of hesitancy about COVID-19 vaccines, ranging between Egypt (46%), Jordan (25.5%), Malta (25.3%), the USA (19.3%), and India (10.6%) [21,40,41,42,43].

As representatives of the healthcare student population, dental students have a crucial role in disseminating robust information about COVID-19 vaccines’ effectiveness and safety [6]. This social role is backed by the prevailing evidence on healthcare professionals’ impact on shaping public opinion regarding health issues, including vaccination [8,9]. Dental students are also threatened by an array of occupational infections due to their clinical training; therefore, compulsory vaccination strategies for pre-clinical students had been increasingly implemented in recent years [15]. Although positive attitudes are predicted from dental students towards vaccines as they are used to receiving them, they demonstrated suboptimal levels of knowledge and attitudes towards new vaccines [18,19].

In the current study, the economic level was a significant determinant of dental students’ VH, as 37.5% of low-income, 27.8% of low-middle-income, 25.2% of upper-middle-income, and 11.1% of high-income economies students were hesitant about COVID-19 vaccines. This socioeconomic gradient of VH has been recently found among population groups in Italy, where perceived levels of economic hardship were significantly associated with VH [22]. However, there is a lack of evidence on the relationship between the economic level and vaccines acceptance from a global perspective; a recent systematic review showed that the highest acceptance level among healthcare workers was in a high-income country, Israel (78.1%), while the lowest level was in a low-income country, the Democratic Republic of the Congo (27.7%) [44]. Regardless of their methodological heterogeneity, cross-sectional studies showed that acceptance levels of medical students in UMHI countries such as Italy (86.1%) and Poland (92%) were much higher than LLMI countries such as Egypt (35%) [40,45,46].

The socioeconomic gradient of VH can be better understood in light of the contextual, individual, and vaccine-specific drivers endorsed by the WHO SAGE. The students of UMHI countries had significantly lower levels of dependence on media/social media (30.4% vs. 42%; *p* < 0.01), were less influenced by public figures (14.5% vs. 21.3%; *p* < 0.01), had less mistrust of government (30.1% vs. 40.6%; *p* < 0.01), less mistrust of the pharmaceutical industry (19.4% vs. 28.9%; *p* < 0.01), less readiness to reject vaccines based on personal/cultural/religious values (10.9% vs. 18%; *p* = 0.003), and less misconception about natural immunity (22.4% vs. 38.1%; *p* < 0.01) than their colleagues of LLMI countries. Contrarily, the students of UMHI countries had significantly higher levels of sufficient knowledge (33.1% vs. 27%; *p* < 0.01), knowledge of vaccine risk/benefit ratio (54.4% vs. 40.2%; *p* < 0.01), willingness to take new vaccines (45.2% vs. 37.6%; *p* < 0.01), and confidence about local availability of vaccines (43.2% vs. 33.6%; *p* < 0.01).

Almost 70% of our sample was composed of female students, and this represents the actual female/male ratio of dental students globally. According to the latest report of the Council of European Dentists (CED), the dental profession is majorly practised by females in Europe [47]. The Health Policy Institute (HPI) of the American Dental Association (ADA) revealed that female dental practitioners had increased from 16% in 2001 to 34.5% in 2020, and this pattern is projected to continue given the steady increase in female dental students from 11.7% in 1979 to 51.6% in 2019 [48,49]. The global phenomenon of “feminization of dentistry” is profoundly evident in low-income settings, including Central Asia, Africa, and South America [50,51,52].

Female dental students had a statistically significant higher level of COVID-19 VH (*χ2* = 9.18; *p* = 0.02) than their male colleagues. This finding is in agreement with the recent systematic review of Sallam (2021) on COVID-19 vaccines, which revealed that males were more likely to accept vaccines [44]. In both LLMI and UMHI countries, female students had higher levels of VH (32% and 20.3%, respectively) than their male colleagues (25.6% and 18.3%, respectively). The emerging evidence on COVID-19 VH among general population groups shows that females are less accepting for COVID-19 vaccines in UMHI countries such as the US, United Kingdom (UK), Portugal, Jordan, Kuwait, and Saudi Arabia and LLMI countries such as Nigeria, Bangladesh, and Ethiopia [21,53,54,55,56,57,58].

Pre-clinical dental students had a statistically significant higher level of COVID-19 VH (*χ2* = 7.39; *p* = 0.007) than their clinical colleagues. Lucia et al. (2020) found that clinical medical students (62%) were significantly more inclined to participate in vaccine trials than their pre-clinical colleagues (44%) in Michigan, USA [59]. This pattern was also found among medical students in India and Poland and dental students in the USA [43,46,60]. Apart from COVID-19 vaccines, the clinical medical students had higher acceptance levels of the influenza vaccine in Brazil, Germany and Saudi Arabia, and the human papillomavirus vaccine in India than their pre-clinical colleagues [61,62,63,64]. One reason to explain this is what Wicker et al. found in their 2013 study, where clinical students had higher levels of perceived occupational risk than pre-clinical students due to their proximity to blood- and air-borne infections; thus, explaining their increased likelihood to accept vaccination [64].

Previous infection with SARS-CoV-2 might be a barrier for vaccination, as the students in our study who recovered from COVID-19 were considerably more resistant (20.4% vs. 12.6%) and slightly more hesitant (24.1% vs. 22.2%) to be vaccinated than their colleagues who were not infected. This finding confirms the results of a recent Italian cross-sectional study, where the recovered COVID-19 patients showed substantial levels of VH towards the COVID-19 vaccine (59.2%) and influenza vaccine (54.6%) [23]. Similarly, providing care to COVID-19 patients was also a barrier for vaccination, as the students who looked after COVID-19 patients were considerably more resistant (18.2% vs. 12.3%) and slightly more hesitant (23% vs. 22.3%) about vaccination. This can be explained by the fact that more than one-third of the students in our sample who provided care to COVID-19 patients had a high level of misconception about immunization.

Using media and social media as the primary source of vaccine-related information was found to be another barrier to be willing to be vaccinated for dental students, as VH levels were significantly higher among the students who used media and social media in both UMHI countries and LLMI countries as their primary source of information. Li Ho et al. (2020) found that 27.5% of the most popular videos about COVID-19 on YouTube contained misleading information; thus, necessitating urgent interventions by health organizations to work proactively with content creators to disseminate high-quality videos [65]. The vaccine opposing content on Twitter was steadily increasing during the first half of 2020; thus, requiring thematic analysis of such content [66]. The discourse analysis of Griffith et al. (2021) for COVID-19 VH tweets found that concerns over safety, mistrust of governments and drug manufacturers, and insufficient knowledge about vaccines were the critical drivers of VH [67].

Trust in governments and their capacity to provide the best available vaccine was a significant suppressor of VH among dental students, as VH was reported by only 11.3% of the students who trusted their governments compared to 29.7% of the students who did not trust their governments. One of the lessons learned from the swine flu (H1N1) pandemic of 2009 was that vaccine acceptance levels during contagious outbreaks are prominently influenced by public confidence in the governments’ technical and organizational skills [68]. Similarly, trust in the pharmaceutical industry had a promoter role in COVID-19 vaccine acceptance, as VH was reported by only 11% of the students who trusted the pharmaceutical industry compared to 33% of the students who did not trust it. In the recent meta-analysis of Díaz Crescitelli et al. (2020), the lack of trust in the pharmaceutical industry was found to be triggered by suspicions about their financial interests and their relationships with governments [69].

The perceived level of knowledge sufficiency about vaccines and their effectiveness was a significant determinant of COVID-19 vaccine acceptance among dental students globally. Male students had higher levels of sufficient knowledge than their female colleagues (39.8% vs. 28.3%; *p* < 0.01), and UMHI students were slightly more knowledgeable than LLMI students (33.1% vs. 27%; *p* < 0.01). VH was reported by 10.8% of the students with sufficient knowledge, while it was reported by 32.8% of the students with insufficient knowledge. For a better understanding of the role of perceived knowledge in vaccine acceptance, the perceived knowledge level was found to be associated with increased awareness about the risk/benefit ratio of COVID-19 vaccination (*U* = 1,719,706.5; *p* < 0.01), a higher level of readiness to take new vaccines (*U* = 1,773,557; *p* < 0.01), and a lower level of misconception about immunization (*U* = 2,808,567; *p* = 0.576). Therefore, the results of this study call for urgent and further implementation of the epidemiology of infectious diseases education and vaccination trends within undergraduate dental curricula for better preparation of dental students for future outbreaks [70,71,72,73].

Availability of vaccines was a significant promoter for COVID-19 vaccines acceptance among dental students globally, as the students who believed that COVID-19 vaccines would be available for them were less hesitant (28.1% vs. 14.4%; *p* < 0.01). In the USA, COVID-19 vaccine acceptance levels increased gradually over the last months, which indicates that increasing trends of vaccine acceptance may correlate positively with the shift from hypothetical vaccines to the availability of real-world vaccines with substantial effectiveness and safety [74]. In the UMHI group, Italy was the most accepting country (4.7 ± 0.8), while Russia was the least accepting country (2.85 ± 1.12). On 1st March 2021, 5.08% of the Italian population and only 2.48% of the Russian population received at least one dose of COVID-19 vaccines [75]. In the LLMI group, Pakistan was the most accepting country (3.97 ± 1.01), while Tunisia was the least accepting country (2.88 ± 1.2). On 16th March 2021, 0.6% of the Pakistani population and 0% of the Tunisian population were vaccinated [75].

### 4.1. Study Strengths

To the best of the authors’ knowledge, this is the first study to assess the prevalence of COVID-19 VH among dental students globally. The study also provides coherent evidence on the impact of gender, clinical training, and economic level on the attitudes of dental students towards COVID-19 vaccination. The drivers of VH endorsed by the WHO SAGE were explored for the first time in the population of healthcare students, and they yielded revealing and conclusive results.

### 4.2. Study Limitations

The first limitation of this study is its sample which is not equally distributed across gender, but this can be simply justified as the sample aimed to be as representative as possible. The second limitation is the imbalanced representation of economic levels, and this occurred mainly due to the disproportionate geographic representation of the IADS. Lastly, the study is naturally limited as a cross-sectional study by its snapshot scope; however, VH levels had been dynamically changing over time, requiring ideally prospective cohort studies to monitor these trends.

### 4.3. Study Implications

Future research on VH among healthcare students should explore the role of university curricula on students’ knowledge about vaccine effectiveness and safety. A conceptual model for the drivers of attitude towards vaccination among healthcare students, including dental students, should be designed and validated using large datasets, such as our dataset that incorporate various variables of interest. Moreover, future research will benefit from prospective cohort studies as they can record the dynamics of attitudes towards vaccines influenced by the emerging achievements and disruptions. The undergraduate dental curricula have to functionally integrate components on infectious diseases epidemiology, especially air-borne infections and vaccination trends, as part of their dental public health and infection control modules. Dental education needs to empower students with sufficient knowledge for making them ambassadors of high-quality health information and to spread the culture of trusting science.

## 5. Conclusions

The overall acceptance level (63.5%) of dental students for COVID-19 vaccines worldwide was suboptimal. The worrisome level of VH (22.5%) was impacted by the economic context where the dental students live and study, as the students from high-income setting were more confident of receiving COVID-19 vaccines. The male gender and clinical training were also associated with an increased level of vaccines acceptance. The media and social media, public figures, insufficient knowledge about vaccines and their safety, and mistrust of governments and the pharmaceutical industry were barriers for vaccination. The findings of this global study call for further implementation of infectious diseases epidemiology education and vaccination trends within undergraduate dental curricula.

## Figures and Tables

**Table 1:** The results of the test re-test reliability ^1^.

Participant	*κ* Coefficient	Participant	*κ* Coefficient
No. 1	0.8	No. 10	0.6
No. 2	0.8	No. 11	1.0
No. 3	0.9	No. 12	0.7
No. 4	1.0	No. 13	1.0
No. 5	1.0	No. 14	0.8
No. 6	0.6	No. 15	0.7
No. 7	0.6	No. 16	1.0
No. 8	1.0	No. 17	0.8
No. 9	0.6	No. 18	0.8

^1^ Cohen’s Kappa statistic *(κ)*: 0.01–0.2 as none to slight, 0.21–0.4 as fair, 0.41–0.6 as moderate, 0.61–0.80 as substantial, and 0.81–1.0 as perfect agreement [32].

**Table 2:** Demographic characteristics of the participating dental students worldwide, February 2021.

Variable	Outcome	Frequency	Percentage	Cumulative Percentage
Gender	Female	4682	70.5%	70.5%
	Male	1836	27.7%	98.2%
	Non-binary	53	0.8%	99%
	Prefer not to say	68	1%	100%
Age	17–22 years	4218	63.5%	63.5%
	23–40 years	2421	36.5%	100%
Academic Level	1st Year	979	14.7%	14.7%
	2nd Year	1227	18.5%	33.2%
	3rd Year	1422	21.4%	54.6%
	4th Year	1259	19%	73.6%
	5th Year	817	12.3%	85.9%
	6th Year	240	3.6%	89.5%
	Internship	322	4.9%	94.4%
	Fresh Graduate	373	5.6%	100%
Country	Albania	57	0.9%	0.9%
	Canada	183	2.8%	3.6%
	Croatia	169	2.5%	6.2%
	Ecuador	381	5.7%	11.9%
	Estonia	78	1.2%	13.1%
	Indonesia	416	6.3%	19.3%
	Iran	388	5.8%	25.2%
	Iraq	354	5.3%	30.5%
	Italy	492	7.4%	37.9%
	Latvia	129	1.9%	39.9%
	Lebanon	107	1.6%	41.5%
	Lithuania	291	4.4%	45.9%
	Malaysia	350	5.3%	51.1%
	Nepal	153	2.3%	53.4%
	Pakistan	379	5.7%	59.2%
	Palestine	417	6.3%	65.4%
	Portugal	389	5.9%	71.3%
	Russia	596	9.0%	80.3%
	Sudan	467	7.0%	87.3%
	Tunisia	283	4.3%	91.6%
	Turkey	386	5.8%	97.4%
	USA	174	2.6%	100%
Geographic Region	Africa	750	11.3%	11.3%
	Americas	738	11.1%	22.4%
	Asia-Pacific	1298	19.6%	41.9%
	Eastern Mediterranean	1266	19.1%	61%
	Europe	2587	39%	100%
Economic Level	Low-income Economy	467	7%	7%
	Lower-middle-income Economy	1232	18.6%	25.6%
	Upper-middle-income Economy	3035	45.7%	71.3%
	High-income Economy	1905	28.7%	100%

**Table 3:** COVID-19-related experience of the dental students stratified by economic level, February 2021.

Variable	LLMI	UMHI	Total	Sig. ^1^
I had been infected by SARS-CoV-2	332 (19.5%)	773 (15.6%)	1105 (16.6%)	<0.01
I had been caring for someone with COVID-19 infection	538 (31.7%)	1270 (25.7%)	1808 (27.2%)	<0.01
I know someone who had COVID-19 infection	1515 (89.2%)	4286 (86.8%)	5801 (87.4%)	0.01
I know someone who had died from COVID-19 infection	945 (55.6%)	2086 (42.2%)	3031 (45.7%)	<0.01

^1^ Chi-squared test was used with a significance level ≤0.05.

**Table 4:** Dental students’ attitudes towards the COVID-19 vaccine stratified by economic level, February 2021.

Variable	Outcome	LLMI	UMHI	Total	Sig. ^1^
I am willing to take the COVID-19 vaccine once it becomes available to me.	Totally Disagree = 1	150 (8.8%)	341 (6.9%)	491 (7.4%)	0.01
	Disagree = 2	126 (7.4%)	308 (6.2%)	434 (6.5%)	0.09
	Not Sure = 3	517 (30.4%)	977 (19.8%)	1494 (22.5%)	<0.01
	Agree = 4	441 (26%)	1054 (21.3%)	1495 (22.5%)	<0.01
	Totally Agree = 5	465 (27.4%)	2260 (45.7%)	2725 (41%)	<0.01
	Total (1–5)	3.56 ± 1.21	3.93 ± 1.23	3.83 ± 1.24	<0.01

^1^ Chi-squared and Mann–Whitney U-test were used with a significance level ≤0.05.

**Table 5:** Dental students’ vaccine-related attitude drivers stratified by economic level, February 2021.

Variable	Outcome	LLMI	UMHI	Total	Sig. ^1^
Contextual Drivers					
Do reports you hear/read in the media/on social media make you re-consider the choice to take the COVID-19 vaccine?	Yes = 2	713 (42%)	1504 (30.4%)	2217 (33.4%)	
	Not Sure = 1	464 (27.3%)	1055 (21.4%)	1519 (22.9%)	
	No = 0	522 (30.7%)	2381 (48.2%)	2903 (43.7%)	
	Total (0–2)	1.11 ± 0.85	0.82 ± 0.87	0.9 ± 0.87	<0.01
Do celebrities, religious or political leaders influence your decision about being vaccinated?	Yes = 2	362 (21.3%)	716 (14.5%)	1078 (16.2%)	
	Not Sure = 1	227 (13.4%)	600 (21.1%)	827 (12.5%)	
	No = 0	1110 (65.3%)	3624 (73.4%)	4734 (71.3%)	
	Total (0–2)	0.56 ± 0.82	0.41 ± 0.73	0.45 ± 0.76	<0.01
Do you trust that your government is making decisions in your best interest with respect to what vaccines are provided (e.g., your government purchases the highest quality vaccines available)?	Yes = 2	460 (27.1%)	1871 (37.9%)	2331 (35.1%)	
	Not Sure = 1	550 (32.4%)	1580 (32%)	2130 (32.1%)	
	No = 0	689 (40.6%)	1489 (30.1%)	2178 (32.8%)	
	Total (0–2)	0.87 ± 0.81	1.08 ± 0.82	1.02 ± 0.82	<0.01
Do you trust pharmaceutical companies to provide credible data on COVID-19 vaccine safety and the effectiveness of the vaccines?	Yes = 2	629 (37%)	2521 (51%)	3150 (47.4%)	
	Not Sure = 1	579 (34.1%)	1462 (29.6%)	2041 (30.7%)	
	No = 0	491 (28.9%)	957 (19.4%)	1448 (21.8%)	
	Total (0–2)	1.08 ± 0.81	1.32 ± 0.78	1.26 ± 0.79	<0.01
Do you know anyone who will not take the vaccine because of religious or cultural values?	Yes = 2	289 (17%)	1234 (25%)	1523 (22.9%)	
	Not Sure = 1	181 (10.7%)	649 (13.1%)	830 (12.5%)	
	No = 0	1229 (72.3%)	3057 (61.9%)	4286 (64.6%)	
	Total (0–2)	0.45 ± 0.77	0.63 ± 0.86	0.58 ± 0.84	<0.01
If “Yes”, do you agree with these people?	Yes = 2	52 (18%)	135 (10.9%)	187 (12.3%)	
	Not Sure = 1	30 (10.4%)	162 (13.1%)	192 (12.6%)	
	No = 0	207 (71.6%)	937 (75.9%)	1144 (75.1%)	
	Total (0–2)	0.46 ± 0.78	0.35 ± 0.67	0.37 ± 0.69	<0.01
Individual/Group Drivers					
Do you think that there are better ways to prevent COVID-19 than using vaccines (e.g., developing immunity by becoming sick and recovering)?	Yes = 2	647 (38.1%)	1109 (22.4%)	1756 (26.4%)	
	Not Sure = 1	432 (25.4%)	1523 (30.8%)	1955 (29.4%)	
	No = 0	620 (36.5%)	2308 (46.7%)	2928 (44.1%)	
	Total (0–2)	1.02 ± 0.86	0.76 ± 0.8	0.82 ± 0.82	<0.01
Do you feel you have enough information about COVID-19 vaccines and their safety?	Yes = 2	458 (27%)	1633 (33.1%)	2091 (31.5%)	
	Not Sure = 1	399 (23.5%)	1439 (29.1%)	1838 (27.7%)	
	No = 0	842 (49.6%)	1868 (37.8%)	2710 (40.8%)	
	Total (0–2)	0.77 ± 0.85	0.95 ± 0.84	0.91 ± 0.845	<0.01
Vaccine-specific Drivers					
Do you think that the benefits of COVID-19 vaccines outweigh their reported side effects/ adverse reactions?	Yes = 2	683 (40.2%)	2686 (54.4%)	3369 (50.7%)	
	Not Sure = 1	661 (38.9%)	1421 (28.8%)	2082 (31.4%)	
	No = 0	355 (20.9%)	833 (16.9%)	1188 (17.9%)	
	Total (0–2)	1.19 ± 0.76	1.38 ± 0.76	1.33 ± 0.76	<0.01
In general, when a new vaccine is introduced, are you inclined to consent to your vaccination?	Yes = 2	638 (37.6%)	2233 (45.2%)	2871 (43.2%)	
	Not Sure = 1	556 (32.7%)	1606 (32.5%)	2162 (32.6%)	
	No = 0	505 (29.7%)	1101 (22.3%)	1606 (24.2%)	
	Total (0–2)	1.08 ± 0.82	1.23 ± 0.79	1.19 ± 0.8	<0.01
Do you feel confident that the health centre or doctor’s office will have the COVID-19 vaccines you need, when you need them?	Yes = 2	571 (33.6%)	2132 (43.2%)	2703 (40.7%)	
	Not Sure = 1	617 (36.3%)	1541 (31.2%)	2158 (32.5%)	
	No = 0	511 (30.1%)	1267 (25.6%)	1778 (26.8%)	
	Total (0–2)	1.04 ± 0.8	1.18 ± 0.81	1.14 ± 0.81	<0.01

^1^ Mann–Whitney U-test was used with a significance level ≤0.05.

**Table 6:** Dental students’ demographic determinants of COVID-19 acceptance, February 2021.

Variable	Outcome	Acceptance Level	Sig. ^1^
Gender	Female	3.83 ± 1.23	0.01
	Male	3.87 ± 1.26	
	Non-binary	3.47 ± 1.42	
	Prefer not to say	3.44 ± 1.43	
Academic Level	1st Year	3.73 ± 1.28	0.11
	2nd Year	3.81 ± 1.19	
	3rd Year	3.91 ± 1.19	
	4th Year	3.90 ± 1.25	
	5th Year	3.93 ± 1.25	
	6th Year	3.80 ± 1.31	
	Internship	3.59 ± 1.26	
	Fresh Graduate	3.66 ± 1.26	
Clinical Training	Preclinical (1st year and 2nd year)	3.77 ± 1.32	0.01
	Clinical (3rd year–Graduate)	3.86 ± 1.24	
Country	Albania	3.14 ± 1.27	<0.01
	Canada	4.41 ± 1	
	Croatia	3.73 ± 1.26	
	Ecuador	3.86 ± 1.19	
	Estonia	3.62 ± 1.22	
	Indonesia	4.37 ± 0.89	
	Iran	3.38 ± 1.38	
	Iraq	3.27 ± 1.17	
	Italy	4.7 ± 0.8	
	Latvia	3.8 ± 1.37	
	Lebanon	3.86 ± 1.18	
	Lithuania	4.18 ± 1.12	
	Malaysia	4.33 ± 0.91	
	Nepal	3.91 ± 1.04	
	Pakistan	3.97 ± 1.01	
	Palestine	3.72 ± 1.27	
	Portugal	4.57 ± 0.85	
	Russia	2.85 ±1.12	
	Sudan	3.37 ± 1.17	
	Tunisia	2.88 ± 1.2	
	Turkey	3.99 ± 1.11	
	USA	4.53 ± 1.04	
Geographic Region	Africa	3.18 ± 1.2	<0.01
	Americas	4.15 ± 1.15	
	Asia-Pacific	4.19 ± 1	
	Eastern Mediterranean	3.5 ± 1.29	
	Europe	3.91 ± 1.26	
Economic Level	Low-income Economy	3.37 ± 1.17	<0.01
	Lower-middle-income Economy	3.63 ± 1.23	
	Upper-middle-income Economy	3.66 ± 1.25	
	High-income Economy	4.36 ± 1.07	

^1^ Mann–Whitney U and Kruskal–Wallis tests were used with a significance level ≤0.05.

**Table 7:** Dental students’ COVID-19 vaccine acceptance and COVID-19-related experience, February 2021.

Variable	No	Yes	Sig. ^1^
I had been infected by SARS-CoV-2	3.88 ± 1.21	3.57 ± 1.36	<0.01
I had been caring for someone with COVID-19 infection	3.89 ± 1.21	3.68 ± 1.31	<0.01
I know someone who had COVID-19 infection	3.68 ± 1.25	3.86 ±1.24	<0.01
I know someone who had died from COVID-19 infection	3.89 ± 1.22	3.77 ± 1.26	<0.01

^1^ Mann–Whitney U-test was used with a significance level ≤0.05.

**Table 8:** Drivers of dental students’ COVID-19 vaccine acceptance worldwide, February 2021.

Category	Driver	No	Yes	Sig. ^1^
Contextual	Media	3.99 ± 1.31	3.71 ± 1.21	<0.01
	Public Figures	3.87 ± 1.25	3.82 ± 1.27	0.24
	Government	3.37 ± 1.30	4.29 ± 1.10	<0.01
	Pharmaceuticals	3.06 ± 1.30	4.31 ± 1.06	<0.01
	Values	4.30 ± 1.05	2.75 ± 1.44	<0.01
Individual/Group	Natural Immunity	4.16 ± 1.11	3.35 ± 1.38	<0.01
	Sufficient Knowledge	3.48 ± 1.19	4.21 ± 1.23	<0.01
Vaccine-specific	Risk/Benefit Ratio	3.14 ± 1.37	4.31 ± 1.06	<0.01
	New Vaccine	3.06 ± 1.28	4.37 ± 1.03	<0.01
	Availability	3.44 ± 1.3	4.17 ± 1.15	<0.01

^1^ Mann–Whitney U-test was used with a significance level ≤0.05.

## Data Availability

The data that support the findings of this study are available from the corresponding author upon reasonable request.

## Supplementary Materials

The following are available online at https://www.mdpi.com/article/10.3390/vaccines9060566/s1, Table S1: Global Questionnaire of Dental Students’ Attitudes towards COVID-19 Vaccines.docx; Table S2: Questionnaire of Dental Students’ COVID-19 Vaccine Hesitancy.

## Appendix A. Members of IADS-SCORE Consortium

- Faculty of Dentistry, McGill University, Canada (Jacques Jaar); Schulich School of Medicine & Dentistry, Western University, Canada (Nima Lighvan); Faculty of Dentistry, University of British Columbia, Canada (Karen Lin);
- Faculty of Dentistry, SEGi University, Malaysia (Sandy Tan Qing Wen); Faculty of Dentistry, University of Malaya, Malaysia (Tan Hian Wei);
- School of Dentistry, Kathmandu University School of Medical Sciences, Nepal (Nitesh Singh);
- Department of Oral and Maxillofacial Surgery, School of Dentistry, Zanjan University of Medical Sciences, Iran (Parsa Firoozi); Faculty of Dentistry, Islamic Azad University Isfahan, Iran (Mohammad Mostafa Aghamohseni; Samin Sirous);
- Institute of Dentistry, CMH Lahore Medical College and Institute of Dentistry, Pakistan (Aneeqa Aslam; Maha Sohail); Dental College, Akhtar Saeed Medical and Dental College, Pakistan (Mehroz Ahmad Khan);
- Faculty of Dentistry, Beirut Arab University, Lebanon (Julien Issa; Mirna Abou Ibrahim);
- Faculty of Dental Medicine, Catholic University of Portugal, Portugal (António Coimbra Amaral);
- Faculty of Dental Sciences, Aldent University, Albania (Ersid Domnori);
- Faculty of Odontology, Universidad San Francisco De Quito, Ecuador (Jorge Ayala); Faculty of Odontology, Catholic University of Cuenca, Ecuador (Maria Sol Medina);
- Faculty of Dentistry, Universitas Indonesia, Indonesia (Viandra Tjokroadiredjo; Farih Aminah);
- Department of Dentistry, Al-rafidain University College, Iraq (Noor Sarmad); College of Dentistry, Uruk University, Iraq (Nabaa Abduladheem); Department of Dentistry, Al-rasheed University College, Iraq (Batool Mohammed);
- Department of Neuroscience, Reproductive Sciences and Dentistry, University of Naples Federico II, Italy (Matteo Cafasso); Department of Biomedical, Surgical and Dental Sciences, School of Dentistry, University of Milan, Italy (Gregorio Tortora); Department of Medicine and Surgery, School of Dentistry, University of Insubria, Italy (Anita Homayuni);
- Faculty of Dentistry, Rīga Stradiņš University, Latvia (Kristīne Romanovska);
- Faculty of Odontology, Medical Academy, Lithuanian University of Health Sciences, Lithuania (Kristė Trijonytė; Julius Mikonis);
- Faculty of Dentistry, University of Khartoum, Sudan (Ahmed Abdalla); Faculty of Dentistry, Al Neelain University, Sudan (Zeinab Hassan); Faculty of Dentistry, University of Medical Sciences and Technology, Sudan (Aya Abdelrahim);
- Faculty of Dental Medicine, Monastir University, Tunisia (Haythem Ben Hadj Belgacem; Maya Fedhila);
- Faculty of Dentistry, Istanbul University, Turkey (İrem Erdoğdu); Faculty of Dentistry, Beykent University, Turkey (Berk Koparan); Faculty of Dentistry, Yeditepe University, Turkey (Ezgi Yeşïltan); Faculty of Dentistry, Marmara University, Turkey (Serap Beşïroğlu);
- Faculty of Dentistry, Ural State Medical University, Russia (Tatiana Spitsyna);
- Faculty of Dental Medicine, University of Rijeka, Croatia (Valentina Mar-asović; Elizabeta Vrkljan; Lovre Labura);
- Institute of Dentistry, Faculty of Medicine, University of Tartu, Estonia (Estelle Saavaste);
- College of Dentistry, University of Florida, United States of America (Natalie Atyeo); School of Dentistry, University of Michigan, United States of America (Alexandra Herzog);
- Oral Health Research and Promotion Unit, Faculty of Dentistry, Al-Quds University, Palestine (Mayar Danadneh);
