# Supplementary material for: Global Prevalence and Drivers of Dental Students’ COVID-19 Vaccine Hesitancy
Source: Vaccines (Basel). 2021 May 29;9(6):566. doi: 10.3390/vaccines9060566 (PMC8226539; doi:10.3390/vaccines9060566)
Supplement: Supplementary file 1 [file vaccines-09-00566-s001.zip › vaccines-1216798-supplementary.pdf]

Table 1: Sample size of participating countries

Country	Target Population	Sample Size
the Republic of Albania	746	254
Canada	1200	300
the Republic of Croatia	1000	278
the Republic of Ecuador	5000	357
the Republic of Estonia	132	98
the Republic of Indonesia	15000	375
the Islamic Republic of Iran	8000	367
the Republic of Iraq	2436	332
the Republic of Italy	5500	359
the Republic of Latvia	530	223
the Lebanese Republic	900	269
the Republic of Lithuania	807	260
Malaysia	3645	348
the Federal Democratic Republic of Nepal	200	132
the Islamic Republic of Pakistan	14800	374
the State of Palestine	3640	347
the Portuguese Republic	3000	341
the Russian Federation	60000	382
the Republic of the Sudan	9000	368
the Republic of Tunisia	1200	291
the Republic of Turkey	29208	379
the United States of America	26000	382

Table 2: Questionnaire of Dental Students' COVID-19 Vaccine Hesitancy

1. **Demographic Data**	
**I.** Gender	• Female<br>• Male<br>• Non-binary<br>• Prefer not to say
**II.** Age	• Dropdown menu of numbers (18-40)
**III.** Academic Level	• 1st Year<br>• 2nd Year<br>• 3rd Year<br>• 4th Year<br>• 5th Year<br>• 6th Year<br>• Intern<br>• Postgraduate
**IV.** Country	• Albania<br>• Burkina Faso<br>• Canada

	• Croatia<br>• Ecuador<br>• Estonia<br>• Germany<br>• Indonesia<br>• Iran<br>• Iraq<br>• Italy<br>• Latvia<br>• Lebanon<br>• Lithuania<br>• Malaysia<br>• Nepal<br>• Nigeria<br>• Pakistan<br>• Palestine<br>• Portugal<br>• Russia<br>• Sudan<br>• Tunisia<br>• Turkey<br>• United States of America
**2. COVID-19-related Experience**	
**V.** I had been infected by SARS-CoV-2	• Yes<br>• No
**VI.** I had been caring for someone with COVID-19 infection	• Yes<br>• No
**VII.** I personally know someone who had died from COVID-19 infection	• Yes<br>• No
**VIII.** I personally know someone who had COVID-19 infection	• Yes<br>• No
**3. Willingness to take COVID-19 Vaccine**	
**IX.** I am willing to take the COVID-19 vaccine once it becomes available to me.	• Totally Disagree<br>• Disagree<br>• Not Sure<br>• Agree<br>• Totally Agree
**4. Drivers of COVID-19 vaccine-related Attitude**	
**X.** Do reports you hear/read in the media/ on social media make	• Yes<br>• No<br>• Not sure

you re-consider the choice to take COVID-19 vaccine?	
**XI.** Do celebrities, religious or political leader influence your decision about getting vaccinated?	• Yes<br>• No<br>• Not sure
**XII.** Do you know anyone who does not take a vaccine because of religious or cultural values?	• Yes<br>• No<br>• Not Sure
**XIII.** If "Yes", Do you agree or disagree with these persons?	• Yes<br>• No<br>• Not sure
**XIV.** Do you trust that your government is making decisions in your best interest with respect to what vaccines are provided (e.g., your government purchases the highest quality vaccines available)?	• Yes<br>• No<br>• Not sure
**XV.** Do you trust pharmaceutical companies to provide credible data on COVID-19 vaccine safety and effectiveness vaccines?	• Yes<br>• No<br>• Not sure
**XVI.** Do you think that there are better ways to prevent diseases than using COVID-19 vaccines (e.g., developing immunity by getting sick and recovered)?	• Yes<br>• No<br>• Not sure
**XVII.** Do you feel you have enough information about COVID-19 vaccines and their safety?	• Yes<br>• No<br>• Not sure
**XVIII.** Do you think that the benefits of COVID-19 vaccines outweigh their reported side effects / adverse reactions?	• Yes<br>• No<br>• Not sure
**XIX.** In general, when a new vaccine is introduced, are you inclined to consent on your vaccination?	• Yes<br>• No<br>• Not sure
**XX.** Do you feel confident that the health centre or doctor's office will have the COVID-19	• Yes<br>• No<br>• Not sure

vaccine you need, when you need them?	
